# Early Attention to Animacy: Change-Detection in 11-Month-Olds

**DOI:** 10.1177/14747049211028220

**Published:** 2021-06-28

**Authors:** Ruth Hofrichter, Hasan Siddiqui, Marcus N. Morrisey, M. D. Rutherford

**Affiliations:** 1 Department of Psychology, Neuroscience and Behaviour, McMaster University. Hamilton, Ontario, Canada

**Keywords:** animacy, change-detection, change-blindness, social attention

## Abstract

Adults are faster and more accurate at detecting changes to animate compared to inanimate stimuli in a change-detection paradigm. We tested whether 11-month-old children detected changes to animate objects in an image more reliably than they detected changes to inanimate objects. During each trial, infants were habituated to an image of a natural scene. Once the infant habituated, the scene was replaced by a scene that was identical except that a target object was removed. Infants dishabituated significantly more often if an animate target had been removed from the scene. Dishabituation results suggested that infants, like adults, preferentially attend to animate rather than to inanimate objects.

Humans are efficient at detecting animate agents and attend to them preferentially (New et al., 2007; Ro et al., 2007). Animacy detection is the ability to quickly distinguish between what is animate and what is inanimate. Animacy detection was functional and allowed for fast identification of social partners and potential predators and prey in the environment of evolutionary adaptedness. It is assumed that selection pressures have facilitated the evolution of both efficient detection of animate objects and selective attention to animate stimuli. This attention is recruited spontaneously, regardless of the context and current goals of the observer (New et al., 2007), and is irresistible (Scholl & Gao, 2013). Selective attention to animacy is not exclusive to humans but apparent across species. Newborn chicks, like humans, show an attunement to animacy (Regolin et al., 2000; Salva et al., 2015; Vallortigara, 2012). Failure to detect a potential predator could have resulted in injury or death, providing a selection pressure for the detection of animate stimuli (Öhman et al., 2001). Attention to humans, of course, was particularly relevant as they were potential friends, foes, or mating opportunities.

## Early Attention to Animacy

Preferential attention to humans and other animate objects develops early. Newborn infants prefer looking at face-like stimuli compared to inanimate objects and use the same cortical routes to process face stimuli as adults would (Buiatti et al., 2019; Fantz, 1963)). Even when stimulus familiarity is controlled for, 9-week-old infants smile and vocalize more toward a person than a doll, indicating that they recognize which one is a social partner (Legerstee et al., 1987). By 12 weeks of age, infants look longer at a person than at a toy monkey (Brazelton et al., 1974).

This sensitivity to animacy is not only based on appearance; motion alone also acts as a cue to animacy. Newborn infants can already distinguish between animate and inanimate motion (Di Giorgio et al., 2017 , 2021) and infants show a looking time preference for animate motion (Frankenhuis et al., 2013; Rochat et al., 1997). Likewise, newborn chicks preferentially attend to animate agents versus inanimate motion (Lorenzi et al., 2017; Rosa-Salva et al., 2018). Infants are sensitive to animacy cues such as self-propulsion (Di Giorgio et al., 2017; Mascalzoni et al., 2010), expect animate agents to act rationally (Csibra et al., 1999), in a goal-directed manner (Wagner & Carey, 2005), and to adhere to the laws of physics (Arterberry & Bornstein, 2002; Spelke, Phillips, & Woodward, 1995). Seven-month-old infants are surprised if objects move on their own but not when people do (Träuble et al., 2014; Woodward et al., 1993). This indicates that within the first year of life, infants are not only interested in animate stimuli but develop a more sophisticated understanding of what stimuli have agency and which do not.

When it comes to imitation, Meltzoff reported that 18-month-olds were six times more likely to complete actions modelled by a human than those modelled by a robot (Meltzoff, 1995). Legerstee and colleagues replicated this finding with 10-month-old infants (Legerstee & Markova, 2008). Together, these studies indicate that preverbal infants have already developed a representation of animate beings that combines static attributes with dynamic motion. Looking time preferences for animate stimuli suggest that infants pay particular attention to humans and other animate agents. In investigations with adults, a change-detection paradigm offers even stronger evidence of preferential attention for animate stimuli.

## Measuring Attention to Animacy Using a Change-Detection Paradigm

New and colleagues (2007) used a change-detection paradigm to test whether changes to animate stimuli would be detected faster and more frequently than changes to inanimate stimuli. Adult participants were presented with an image of a complex naturalistic scene (Scene A) for 250 ms which was then masked by a blank screen. Then a second image was presented (Scene A’). Scene A’ was either identical to Scene A, or one object had been removed from the scene. The object that was removed was either animate or inanimate. Scene A and A’ alternated on a loop until participants could identify the change. Results indicated that adults were faster and more likely to detect animate compared to inanimate changes. Participants were more likely to experience change-blindness, failing to detect changes, when the changed object was inanimate. This superior detection of changes in animate stimuli was independent of expertise. New and colleagues (2007) used vehicles as one of the stimulus categories, reasoning that if expertise was driving the effect, they should see an advantage for detecting changes to vehicles. Results showed no such advantage, despite the fact that in our current environment, vehicles pose a potential threat. Participants were not simply attending to entities that moved or could be potentially dangerous.

## Current Study

The current study was designed to test for preferential attention to animate over inanimate objects in 11-month-olds using a change-detection paradigm akin to that used by New and colleagues (2007). Young infants have been shown to be sensitive to animate motion (Frankenhuis et al., 2013; Rochat et al., 1997) but no studies have tested infants’ preferential attention to animate objects by using infants’ ability to detect change in static images. To adapt the change-detection paradigm for use with infants, we used a habituation paradigm. We included 11-month-olds as our participants because infants this age have been shown to be attuned to animacy (Rochat et al., 1997) and are able to sustain attention longer than younger infants.

The stimuli in our study were a subset of the scenes used by New et al. (2007). Participants were habituated to Scene A, then presented with Scene A’. In our study there was always a change between Scene A and A’. Using an eye tracker, we measured whether the child dishabituated to the change, suggesting that the child had noticed a difference between Scene A and A’. We predicted that infants, like adults, would be better at detecting changes to animate compared to inanimate entities, suggesting heightened attention.

## Method

Data collection complied with current APA Ethical Principles of Psychologists and Code of Conduct, and all measures, manipulations and exclusions in the study are disclosed.

### Participants

Thirty-six 11-month-old infants (20 female; *M* = 11.13 months, *SD* = .36) were recruited through an existing database for child participants. Parents reported their children’s ethnicity as Hispanic (two), Asian (four) or Caucasian (30). Parents and their children were compensated for their time with $10. Data of 10 participants was excluded from analyses due to computer/calibration failure (three) children not completing trials (three) or parents pointing out objects in the scenes (four). A sensitivity power analysis indicated that the sample (*N* = 26) had 80% power to detect one-tailed, within-subject differences of *d* = 0.5 or higher. The sensitivity power analysis was conducted using the *pwr* package in R 4.0.0.

### Stimuli

**Figure 1.**
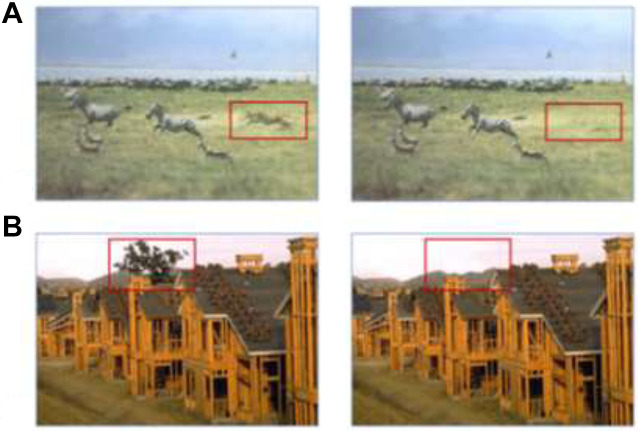
Examples of stimuli with areas of interest highlighted.

The stimuli were color images of complex, natural scenes (see Figure 1 for examples; all stimuli are shown in Appendix A). We selected eight pairs (Scene A & A’) from New et al. (2007) original stimuli set: four pairs associated with changes to animate entities, and four scenes associated with changes to inanimate entities. Animate entities that were removed were a horse, a lion, a man, and an officer. Inanimate entities were a TV, a tree, a cup, and a building (for example in Figure 1A, the lion disappears in Scene A’. In Figure 1B, the tree disappears). Areas of interest (AOI) were created around the target (see Figure 1). We chose images in which the changing element was large so that infants would be more likely to notice the change. The average size of AOIs for animate and inanimate entities was equal (average area of AOI = 0.74 inches). Further, for the inanimate change images, we selected scenes that did not also include any non-changing animate objects because they could potentially distract participants from noticing the inanimate change (Altman et al., 2016).

We defined Habituation as three trials with averaged looking time of less than 50% of the average looking time of the first 3 trials. We also required children to look at the screen for at least three cycles of Scene A before attention dropped to meet habituation criteria. An experimenter who stood beside the infant throughout the experiment noted how many cycles of Scene A the infant attended to and advanced the trial to Scene A’ once the participant had habituated. The eye tracker captured whether the child looked at AOIs. We only included trials in our analysis if infants had looked at the area of interest in Scene A at least once, to ensure that infants had an opportunity to notice the target in Scene A before it was removed in Scene A’.

Dishabituation was noted only if 1) the child had met the habituation criterion, 2) looking time recovered to above 50% of baseline looking time at test and, 3) the child looked at the area of interest in Scene A’, the location of the removed object, at least once.

### Procedure

At the beginning of the session, parents completed a demographic survey providing information on the age, sex, and ethnicity of their infant. To track infants’ eye movements, we used a Tobii T60XL eye tracker (24-inch screen; 1920 × 1080 pixels widescreen monitor). The infant was positioned in front of the eye tracker on the parent’s lap approximately 24 inches away from the screen. Parents wore sunglasses to ensure that their eyes were not detected by the eye tracker.

At the beginning of each trial, an attention-grabber appeared: a video clip of a duck shaped rattle that shakes and makes a loud, quacking sound. The attention-grabber used was one included with the Tobii studio software and was shown continuously until it successfully directed the child’s attention towards the screen. As soon as the child was looking directly at the screen, Scene A was displayed.

**Figure 2.**
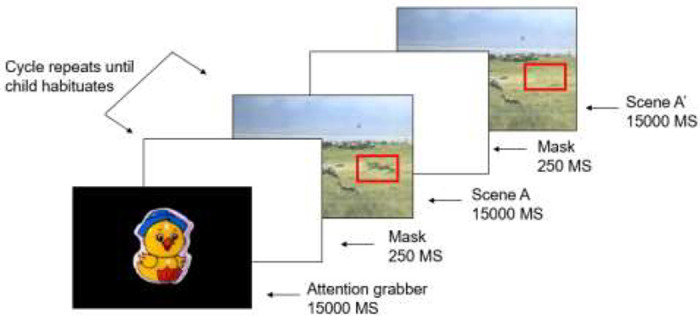
Trial structure.

Scene A was displayed for 15 seconds. After 15 seconds, a blank screen masked the screen for 250 ms, then Scene A returned. This cycle continued until the infant had habituated to Scene A. Scene A was shown a minimum of six times and a maximum of 12 times. Our inclusion criterion was that if a child did not habituate within 12 cycles, the trial would be excluded from analysis. Once habituation was achieved, Scene A was masked again with a blank screen. Lastly, Scene A’ was presented for 15 seconds, then the trial was over (see Figure 2).

## Results

Participants were assigned a score of 0 or 1 for each trial. A score of 1 indicated that they dishabituated at test, while a score of 0 indicated no dishabituation. Every child could receive a maximum of eight scores (four animate trials, four inanimate trials). On average, 11-month-olds completed 3.4 (out of 4) animate (SD = 1.12) and 2.04 (out of 4) inanimate (SD = 1.74) trials. Further, on average, participants watched 6.34 cycles of animate scenes (SD = .55) and 6.23 of inanimate scenes (SD = .51) until habituation was achieved.

### Analytic Strategy

We conducted a mixed-effects linear model to analyze children’s dishabituation rates to animate versus inanimate stimuli. The model included a fixed effect for *Category* (Animate vs. Inanimate trials) and a random intercept for *Subject.* We used a linear model instead of a logistic model as some have argued that linear models may be optimal for analyzing binary responses (Gomila, 2019). Additionally, linear models are less hampered by smaller sample sizes.

We conducted a second mixed-effects linear model to analyze children’s looking time toward animate versus inanimate AOIs. This model also included a fixed effect for *Category* (Animate vs. Inanimate) and a random intercept for *Subject*.^1^ All analyses were conducted using the *lmer* command in the *lme4* package in R 4.0.0. The default optimizer was used.

### Findings

**Figure 3.**
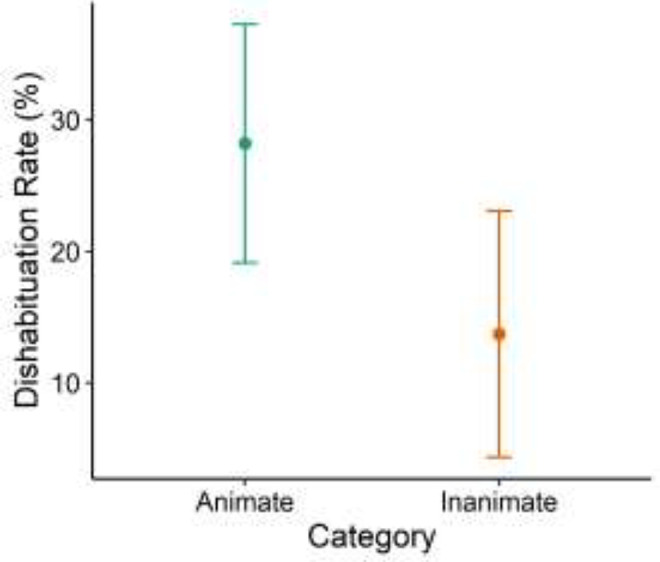
Dishabituation rates for animate versus inanimate changes. Error bars represent 95% confidence intervals.

**Figure 4.**
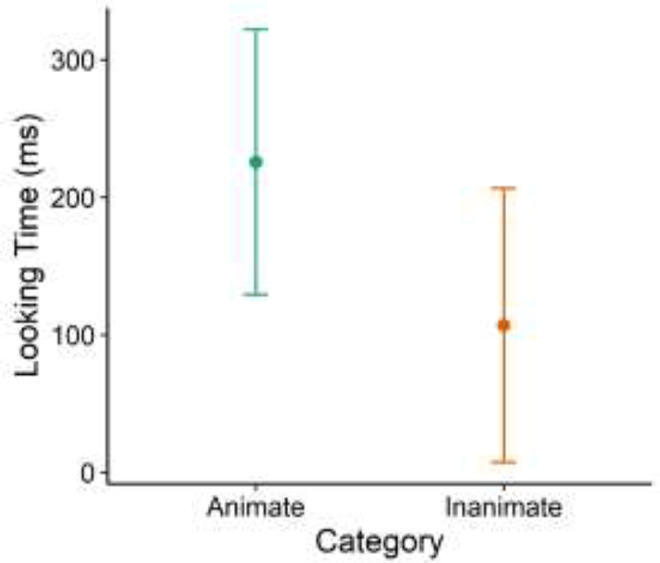
Looking time toward animate versus inanimate areas of interest (AOI). Error bars represent 95% confidence intervals.

There was a significant effect of Category on Dishabituation rates (Wald χ^2^(1) = 4.83, *p* = 0.03, Hedges’ *g* = 0.36, 95% CI [0.04, 0.68]; See Figure 3). Children dishabituated more often to Animate stimuli (*M* = 0.28, *SD* = 0.45) than Inanimate stimuli (*M* = 0.14, *SD* = 0.35). However, there was only a marginal effect of Category on Looking Time (Wald χ^2^(1) = 2.87, *p* = 0.09, Hedges’ *g* = 0.27, 95% CI [−0.05, 0.59]; See Figure 4). Children spent slightly more time looking at Animate stimuli (*M* = 225.48, *SD* = 511.35) than Inanimate stimuli (*M* = 106.81, *SD* = 323.13).

## Discussion

Our results revealed higher dishabituation rates for animate change trials compared to inanimate change trials. Infants were more likely to notice changes if an animate object was removed from the scene compared to an inanimate object, which is consistent with the findings reported by New and colleagues (2007). These results suggest that in the first year of life, infants already have the attentional preference for animate objects that adults do.

New and colleagues (2007) argued that an attentional preference for animate objects had functional significance in our evolutionary past and could not be explained by familiarity. Animate objects in our study included a horse and a lion, animals that infants in our study might have never encountered before. Inanimate objects included objects like a TV or a cup. Since most infants in our study are likely to have seen and interacted with a TV or a cup, familiarity cannot account for our results. Rather, preferences for detecting animate changes early in life add further evidence to New and colleagues’ (2007) theory that preferential attention for animacy is evolutionarily driven. This is in line with evidence that other species, such as chicks, show a preference for animate stimuli from birth onward (Lorenzi et al., 2017; Regolin et al., 2000; Rosa-Salva et al., 2018; Salva et al., 2015; Vallortigara, 2012).

This is the only experiment that we know of that has adapted the adult change-detection paradigm for use with infants. That said, Wang and Baillargeon (2006) used a live puppet show style display with occlusion and covering events to show that 11-month-olds can detect a change in the height of an object during an occlusion but not during a covering event. The authors concluded that infants succeeded in detecting a change to an object’s height only when watching an event in which the infant perceives height to be a relevant variable. They failed to detect the same change in an event where height had not yet been identified by the infant as a relevant variable. Similarly, we found a greater frequency for change detection when the object had functional significance to the infant (an animate object) than when the object did not (an inanimate object).

We did not find significant differences in looking time to animate versus inanimate objects. Based on previous research, we did not have an a priori expectation of looking time differing between animate and inanimate entities. However, previous research on infants’ looking time towards animate versus inanimate moving objects has shown conflicting findings. Newborn infants have been shown to spend more time looking at static images of schematic faces versus random patterns (Fantz, 1963), and infants can distinguish animate from inanimate objects and even bind animacy motion cues to animals (Träuble & Pauen, 2011). While Frankenhuis and colleagues (2013) found that 4- and 10-month-old infants strongly preferred looking at animate motion, Rochat and colleagues (1997) only found this pattern for 3-month-old infants and reported a switch in preferential attention towards animate motion around 5–6 months of age. Rochat and colleagues argued that by 6 months of age, infants readily understood the social contingencies underlying the animate display and therefore spent more time looking at the inanimate display, as if scrutinizing the display for some meaning or pattern. Since infants in our study were shown Scene A for a minimum of 45 seconds during habituation, they had ample time to explore all objects in the image. Even if infants were initially drawn to animate AOIs, after identifying and processing animate objects, they could have then moved on to visually explore other objects. If this were the case, we would not see significant differences in looking time across categories of AOIs.

### Limitations and Future Directions

Many participants became bored easily and some did not complete all eight trials of our study. Using inattentional blindness videos akin to those used by Simons and Chabris (1999), instead of static images might be more interesting to infants. In a future study, children could be habituated to version A of a video and at test, version A’ would be shown with an animate or inanimate entity removed. If infants dishabituated at test, it would indicate that they noticed the change.

Further, while we ensured that AOIs were equal in size between the animate and inanimate category, we did not control for the location of the changing objects. Two of our inanimate AOIs (building and cup) were located at the forefront of the scene. Our animate AOIs tended to be located closer to the back of the scene. Additionally, half of our animate entities were shown in mid-motion. The bias for detecting animate changes could be a result of perceiving these entities in motion. This could present a potential confound and should be controlled for in future studies.

Another possible confound in the current study was low-level differences between images of scenes, such as variance in luminance and complexity. New and colleagues (2007) tested whether such low-level visual characteristics predicted performance. They did not find any significant effects. However, although we used the same stimuli as New and colleagues, we did not control for these low-level characteristics in our analysis, so we cannot say for certain whether they presented a confound in our study. It is possible that adults and infants are affected differently by low-level features. An improved follow-up experiment might employ a between-subject design using the same scenes in both groups with an animate object removed in one group and an inanimate object in the other. One of our aims with the current study was to replicate and expand on the study by New and colleagues (2007), so we used the same stimuli that they had used. However, a study that used the same scenes in the animate and inanimate trials would hold a number of potentially relevant variables equal across trial types, such as location, lighting, subject matter, and implied motion.

## Conclusions

Our results show evidence for an early-developing attentional preference for animate objects. By the age of 11 months, infants dishabituate significantly more often for changes to animate compared to inanimate objects. This finding agrees with adults’ faster and better detection rates for animate changes in New and colleagues’ (2007) change-blindness paradigm. Infants, like adults, seem to prioritize attention to animate entities and this result cannot be explained by familiarity.

## Figures and Tables

**Figure A1.**
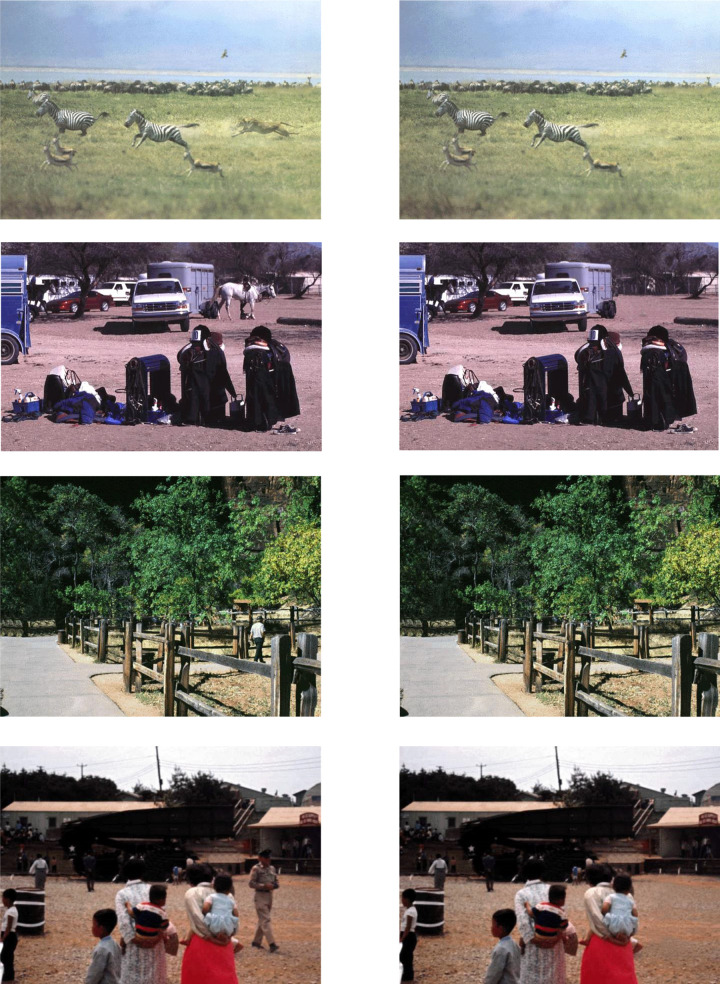
Overview of stimuli set used in this study.

## Note

1. Originally, both models were fitted with a random intercept for *Stimulus* to capture adjustments in the intercept for each scene. However, the random intercept contributed non-significant variance in both models (*p*s > 0.15) so was removed for the final analysis.
